# Incomplete glenosphere seating: a cause for concern

**DOI:** 10.1016/j.xrrt.2026.100668

**Published:** 2026-01-23

**Authors:** Owen J. Rabak, Adrian K. Low

**Affiliations:** a Sydney Local Health District (SLHD), Sydney, Australia; b Specialty Orthopaedics, Norwest Private Hospital, Sydney, Australia

**Keywords:** Reverse total shoulder arthroplasty (rTSA), Glenosphere dissociation, Incomplete seating, Non–morse taper, Central screw backout, Disengagement

Initially developed as a salvage procedure for cuff tear arthropathy, reverse total shoulder arthroplasty (rTSA) has become an increasingly popular surgical option in the treatment of various traumatic and degenerative glenohumeral conditions.^4^ However, with the rapid uptake of rTSA, the total number of complications and revisions have also increased.^6^ Glenosphere–baseplate dissociation was the third most common (12.2%) mode of failure of all rTSA complications reported to the US Food and Drug Administration from 2012-2016.^13^ In the most recent annual report from the Australian Joint Registry, glenoid implant–related failures (dissociation, 3.5%; glenoid implant breakage, 0.9%) were also leading causes for revision rTSA.^9^

Incomplete glenosphere seating may be a prelude to glenoid implant breakage and complete glenosphere–baseplate dissociation. Incomplete seating of the glenosphere can occur for a number of reasons, including soft-tissue interposition, bony impingement, cross threaded screws, technical- and implant-related factors.^3^^,^^8^ In contemporary rTSA designs, the glenosphere is secured to the glenoid baseplate with a central countersunk screw, with or without a peripheral Morse taper. Despite being a commonly reported complication often leading to revision surgery, there are surprisingly only a small number of cases of incomplete glenosphere seating in the published literature, both with Morse taper^3^^,^^7^^,^^11^ and non–Morse taper^5^^,^^12^ designs, describing potential mechanisms of failure and clinical outcomes. Some authors have also reported partial disengagements that are not associated with poor functional outcomes,^11^ as well as the potential for spontaneous reversal of disengagements.^10^ Therefore, the appropriate management of this complication may not always be straightforward and may be dependent on specific implant design.^3^^,^^5^^,^^11^

The purpose of this study was to describe 3 cases of incomplete glenosphere seating in a single-surgeon consecutive series of 130 rTSAs over six years using a contemporary non–Morse taper, central screw–dependent design; to review existing literature on this complication across different fixation mechanisms; and to highlight technical considerations in recognition and management relevant to implant-specific design features.

## Case 1

**Figure 1:**
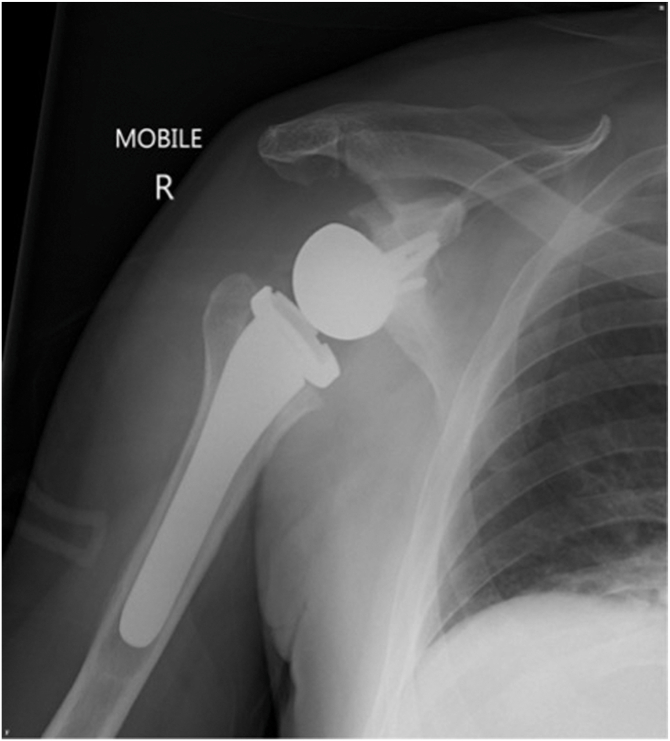
Immediate postoperative radiographs, anteroposterior view.

A 64-year old man underwent rTSA using GPS navigation to address symptoms related to cuff arthropathy of several years duration. There was almost full active shoulder range of movement on clinical examination. A magnetic resonance imaging (MRI) showed a chronic retracted supraspinatus tear and computed tomography (CT) confirmed the presence of cuff arthropathy (Hamada 4a). Unfortunately, his surgery was complicated by a deep periprosthetic infection (*Cutibacterium acnes*) and subsequently proceeded to a two-stage revision, with the first stage about six months following the original surgery. Three months later, the second stage was performed to remove the antibiotic spacer. There was minimal evidence of bone loss and another standard uncemented Equinoxe (Exactech, Gainesville, FL, USA) reverse shoulder arthroplasty implanted with GPS navigation. His immediate postoperative radiograph appeared unremarkable (Fig. 1). The patient made an excellent recovery with normal inflammatory markers and eventually no pain with full active movement in his shoulder, except internal rotation, which was limited to L5.

**Figure 2:**
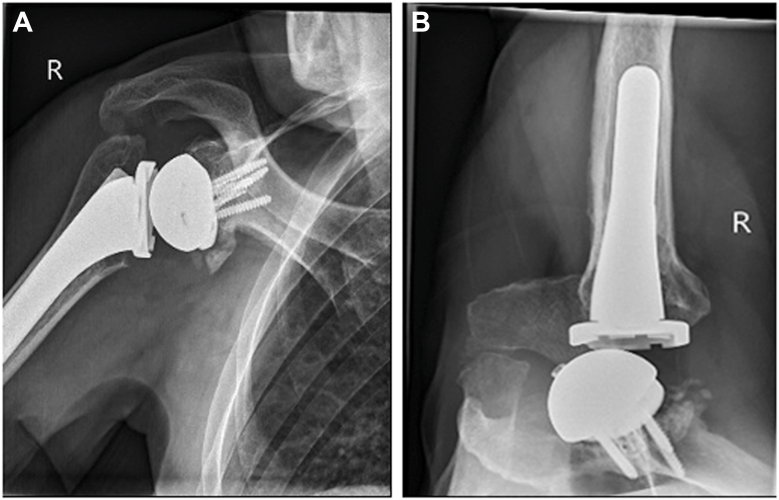
(**A**) 1-year postoperative radiographs, AP. (**B**) 1-year postoperative radiographs, axial. *AP*, anteroposterior.

A year later, the patient developed clicking and squeaking in his shoulder. A radiograph demonstrated a clear step off between glenosphere and baseplate (Fig. 2, *A* and *B*).

**Figure 3:**
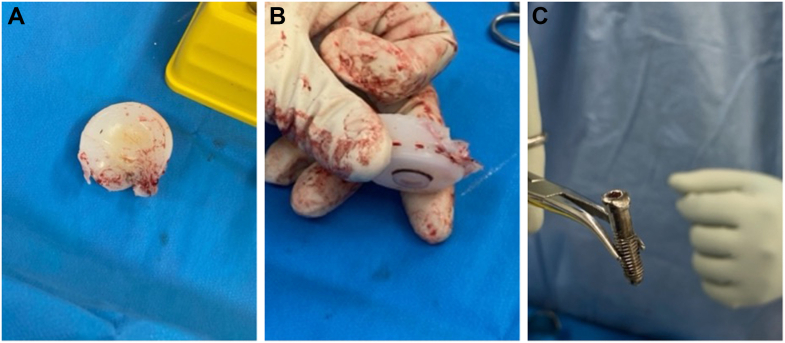
(**A**) Intraoperative findings—destruction of the polyethylene liner. (**B**) Intraoperative findings—destruction of the polyethylene liner. (**C**) Intraoperative findings—deformed central glenosphere locking screw.

As he had no pain and retained full shoulder movement, a period of observation was agreed upon. However, although pain was still not a feature, his squeaking became more prominent (Video 1) over a period of six months, and so proceeded to another revision surgery using the Equinoxe reverse total shoulder system. Intraoperative findings included mild synovial metallosis and catastrophic failure of the polyethylene liner (Fig. 3, *A* and *B*). The glenosphere was still rigidly fixed to the baseplate but the central locking screw had backed out and was deformed (Fig. 3*C*). The central screw could not be removed with a screwdriver, so pliers were used instead. The damaged polyethylene liner was exchanged and a new glenosphere–baseplate construct successfully secured.The patient recovered very well and, four years later, had a pain-free shoulder with full active range of movement.

## Case 2

**Figure 4:**
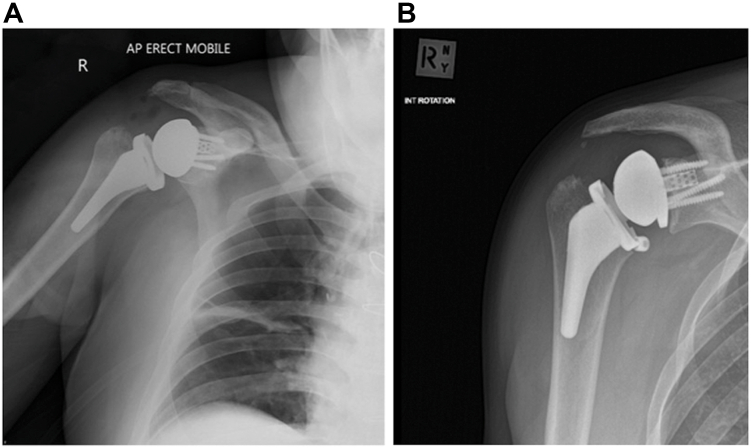
(**A**) Immediate postoperative radiographs, anteroposterior view, with clear stepoff. (**B**) 6-week postoperative radiograph with broken screw head and dissociation of the glenosphere. *AP*, anteroposterior.

A 52-year-old man underwent rTSA for symptomatic cuff arthropathy following a failed revision open rotator cuff repair seven years prior. Pre-operative examination demonstrated global limitation in active and passive shoulder range of movement, with forward elevation to 90°, external rotation to 20°, and internal rotation to the buttock. An MRI showed chronic rupture of all cuff tendons and a CT confirmed advanced cuff arthropathy with superior migration of the humeral head (Hamada 4b). The surgery was uneventful with the central screw seemingly engaged and securely seating the glenosphere. Postoperative radiographs demonstrated a stepoff between baseplate and glenosphere (Fig. 4*A*). The decision was made to observe, and he recovered well from his surgery until six weeks later, when the patient reported sudden new-onset pain, locking, and a clicking sensation in his shoulder. Radiographs now revealed obvious dissociation of the glenosphere and a loose peripheral glenoid locking screw cap sitting inferiorly (Fig. 4*B*).

**Figure 5:**
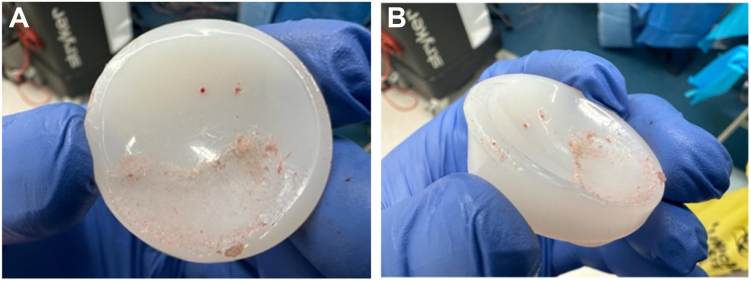
(**A**) Intraoperative findings—damage to polyethylene liner. (**B**) Intraoperative findings—damage to polyethylene liner.

Revision surgery was recommended with intraoperative findings including back out of the central glenosphere-locking screw with complete dissociation of the glenosphere from the baseplate; significant wear of the polyethylene liner (Fig. 5*A* and *B*); a loose peripheral glenoid locking screw cap sitting in the inferior capsule and a humeral stem with no bony ingrowth. A new glenosphere was inserted onto the glenoid baseplate; however, could not be seated flush. Intraoperatively, it became apparent that posterior bony impingement and limited exposure likely contributed to the inability to fully seat the glenosphere during the index procedure. The glenoid was circumferentially reamed, establishing a flat and accessible glenoid surface for complete seating of a new baseplate–glenosphere construct. Finally, the humeral canal was prepared to accept a cemented standard length humeral stem. The patient recovered very well and at 18 months post-operatively had no pain in his shoulder with almost full active range of movement.

## Case 3

A 58-year-old man sustained a minimally displaced 3-part left proximal humerus fracture after being hit by a car and falling off his bicycle. The fracture was managed conservatively but was complicated by osteonecrosis of the humeral head with extensive subchondral collapse and evolving glenohumeral osteoarthritis, confirmed on MRI 18 months later. Eventually, the patient underwent rTSA six years after his original fracture. Preoperative active forward elevation was limited to 150°, external rotation 10°, and internal rotation to the buttock. An MRI showed the cuff tendons to be intact and a CT confirmed advanced glenohumeral osteoarthritis with B1 glenoid morphology. The surgery was uneventful with the central screw seemingly engaged and securely seating the glenosphere.

**Figure 6:**
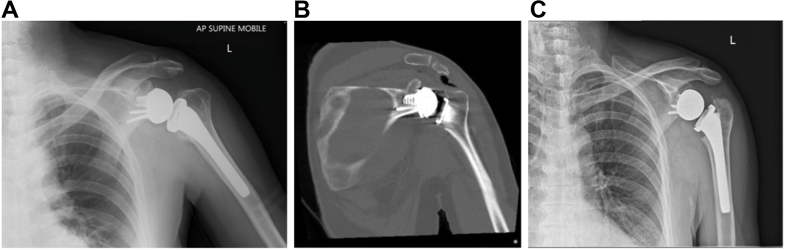
(**A**) Immediate postoperative radiographs, anteroposterior view, with stepoff between glenosphere and baseplate. (**B**) Post-operative CT, coronal view, demonstrating clear stepoff analogous to the radiograph. (**C**) 18-months postoperative radiograph with central screw backout and glenosphere dissociation demonstrated. *AP*, anteroposterior; *CT*, computed tomography.

Immediate post-operative radiographs demonstrated a stepoff between baseplate and glenosphere (Fig. 6*A*). However, the decision was made to observe, as he recovered very well from his surgery, with eventually no pain in his shoulder and almost full active movement, until he represented seven months later with increased activity-related discomfort and restriction in shoulder movement, as well as a clicking sensation in his shoulder. A radiograph and CT at that time demonstrated no further displacement of the glenosphere relative to the baseplate, and as his symptoms did steadily improve again, observation was continued. A progress CT 1 year postsurgery remained unchanged, but an x-ray 18 months postsurgery now showed back out of the central screw as well as osteolysis around the calcar region of the proximal humerus, most likely from polyethylene wear debris (Fig. 6, *B* and *C*). Revision surgery was recommended despite the patient having only minimal discomfort, but active shoulder movement was still limited (forward elevation 100°, external rotation 30°, internal rotation to buttock) with persistent clicking.

**Figure 7:**
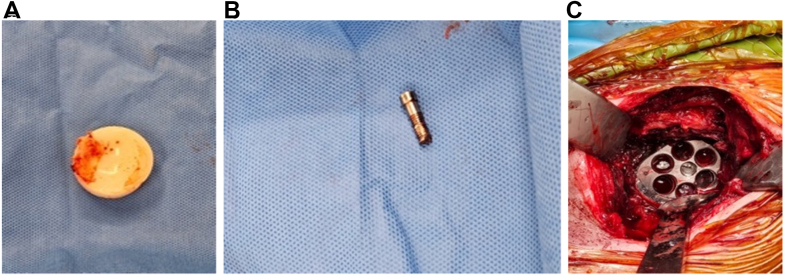
(**A**) Intraoperative findings—damage to polyethylene liner. (**B**) Intraoperative findings—damage to central glenosphere locking screw—baseplate with broken section of central locking screw. (**C**) Intraoperative findings—baseplate with broken section of central locking screw.

Intraoperative findings included mild synovial metallosis, glenosphere loosening, and catastrophic failure of the polyethylene liner (Fig. 7*A*) due to backout of the central glenosphere-locking screw, which had broken flush at the level of glenoid baseplate (Fig. 7, *B*, *C*). The broken central screw remaining within the baseplate, which was likely to be both cross-threaded and cold-welded, was removed together with the baseplate. After removal of the peripheral glenoid screws, the glenoid plate impactor device was applied. Using alternating clockwise-anticlockwise rotational forces to break the bone–implant interface, the baseplate along with the remnant screw, was able to be removed with minimal glenoid bone loss. Another standard baseplate, with an extended cage, was inserted along with a new glenosphere and central screw. There was some osteolysis around the proximal humeral calcar, but the humeral stem was well-fixed. The patient recovered very well and, at 1 year post-operatively, has a pain-free shoulder.

## Discussion

Although a relatively common cause for rTSA revision, outcomes following incomplete glenosphere seating have rarely been reported in the literature.^2^^,^^9^ In this consecutive case series of a contemporary rTSA with a non–Morse taper design, we found an incidence of 2.3% of incomplete glenoid seating, all leading to revision surgery. Using the same prosthesis,^5^ reported 2 cases of incomplete seating (5%) among 40 rTSAs, one partial and one complete disengagement, both requiring revision.^12^ Reported a symptomatic patient with normal radiographs at two years with catastrophic failure of the central screw, leading to glenosphere dissociation and massive polyethylene damage noted at revision surgery.

As well as being non–Morse taper, the Equinoxe system has an oval-shaped baseplate and glenosphere, with the latter designed to sit flush with the baseplate. These design features may avoid the fluid/soft tissue interposition issues and corrosion that can destabilize Morse taper junctions^3^ and may provide better rotational stability than typical circular baseplates. However, it may be more technique-dependent, requiring precise in-line and rotational application of the glenosphere with emphasis on central screw torque and threading integrity to maintain complete glenosphere seating, usually verified intraoperatively with tactile/auditory “squeak” feedback. With meticulous glenoid exposure, soft tissue and bony clearance, and in-line component assembly, the risk of incomplete seating can be minimized, although this complication may still occur, even in experienced practice.

We believe that any situation where glenoid exposure and in-line assembly of components is difficult is at increased risk, including males, stiff shoulders, previous surgery, revisions, and glenoids with significant retroversion. In the current study, all 3 cases of incomplete seating occurred in males, where males represented only 35% of the cohort. These male cases tended to be younger, with greater glenoid deformity and had prior surgery, where adequate exposure and reaming of potentially harder glenoid bone would be even more imperative for complete seating. Though the more recently available “pear-shaped” Equinoxe small baseplate was not utilized in the current cohort, it remains to be seen if the smaller or wedged baseplates, by changing the required angle of approach for component assembly, have the potential to minimize this complication.

On occasions, incomplete glenosphere seating may not have been detected initially. Immediate post-operative radiographs in recovery generally provide only a single view and are usually not tangential to the baseplate. As seen in our study and others,^5^ often patients can be quite asymptomatic for up to several months afterward, but we believe with non–Morse taper designs that rely solely on central screw fixation, eventually there will be fatigue failure of the central screw, resulting in complete glenosphere–baseplate dissociation. Often this is heralded by new-onset symptoms, in particular audible symptoms such as squeaking, clicking, grinding, and clunking, which should alert the surgeon of the possibility for this complication. If revision is delayed, the prominent central screw head may cause significant polyethene liner damage and subsequent osteolysis.^12^ The cases presented in this study also offer some technical tips for handling broken, deformed, cross-threaded or cold-welded screws, and minimizing glenoid loss upon baseplate removal. If incomplete seating remains an issue despite using a new glenosphere and central screw, the baseplate will usually need to removed and the glenoid face reamed to avoid any bony or soft tissue impingement.

It is recognized that different rTSA fixation mechanisms are associated with differing modes of glenosphere disengagement, and current literature suggests that both Morse taper and non–Morse taper designs are susceptible to incomplete seating through distinct mechanical pathways.^1^^,^^13^ Inadequate impaction/engagement of the Morse taper has been identified as a cause of dissociation, which may be related to inadequate reaming of the glenoid, micromotion, implant taper design or manufacturing, and interposed fluid/blood or soft tissues.^3^^,^^8^^,^^11^ Using a Zimmer rTSA system, which relies on Morse taper fixation alone,^10^ one study reported a case of incomplete glenosphere seating with spontaneous reversal after 1 year, suggesting conservative management may be a viable option in low-demand patients. In cases of failed Zimmer reverse shoulder arthroplasty prostheses, 25% of failures were attributable to glenosphere–baseplate dissociation, its most common mode of failure.^13^

## Conclusion

Surgeons should be aware of the potential for this complication, especially in patients who may initially be symptom-free then develop mechanical-type or audible symptoms. Meticulous glenoid exposure and reaming avoiding soft tissue/bony impingement, with in-line and correct rotational assembly of the glenosphere onto the baseplate, can in many cases avoid this complication. Immediate post-operative radiographs performed in recovery are often not adequate, as recognition requires views tangential to the glenoid baseplate. In equivocal or subtle cases, a CT scan should confirm the diagnosis. Surgeons should also be aware of the particular design features of the prosthesis they are utilizing and whether the glenosphere is designed to sit flush with the baseplate or slightly elevated from the bone surface. The key teaching point of this report is that incomplete glenosphere seating may initially be clinically subtle yet progress to mechanical failure, particularly in designs reliant on central screw fixation. Early recognition, appropriate imaging, and timely revision can mitigate polyethylene damage and bone loss, with good functional outcomes following revision surgery. We therefore recommend early revision surgery once the complication is recognized to avoid prolonging suffering and potential for implant- or polyethylene-related bone loss.

## Disclaimers:

Funding: No funding was disclosed by the authors.

Conflicts of interest: The authors, their immediate families, and any research foundation with which they are affiliated have not received any financial payments or other benefits from any commercial entity related to the subject of this article.

Patient consent: Obtained.

## Supplementary Data

Video 1 Case 1: audible squeaking with pain-free shoulder movement.
